# Dermatology Self-Medication in Nursing Students and Professionals: A Multicentre Study

**DOI:** 10.3390/healthcare12020258

**Published:** 2024-01-19

**Authors:** Ana Batalla, Alba-Elena Martínez-Santos, Sara Braña Balige, Sara Varela Fontán, Lucía Vilanova-Trillo, Paz Diéguez, Ángeles Flórez

**Affiliations:** 1 Dermatology Department, University Hospital of Pontevedra, 36162 Pontevedra, Spain; ana.batalla.cebey@sergas.es (A.B.); lucia.vilanova.trillo@sergas.es (L.V.-T.); angeles.florez.menendez@sergas.es (Á.F.); 2 DIPO Research Group, Galicia Sur Health Research Institute (IIS Galicia Sur), SERGAS-UVIGO, 36312 Vigo, Spain; 3 Department of Psychiatry, Radiology Public Health, Nursing and Medicine, Faculty of Nursing, University of Santiago de Compostela, 15782 Santiago, Spain; 4 School of Nursing of the Provincial Council of Pontevedra, University of Vigo, 36004 Pontevedra, Spain

**Keywords:** nurses, students, nursing, self-medication, skin diseases, dermatology, health knowledge, attitudes, practice

## Abstract

Current evidence shows that the prevalence of self-medication in healthcare professionals and their students is troublingly high despite them knowing the risks involved. There is limited research on self-medication in dermatology, and there are even fewer studies on this practice among nurses and nursing students, despite the potential mucocutaneous health problems that may affect them. The aims of our study were to examine the prevalence of self-medication mainly in the field of dermatology among nurses and nursing students as well as to explore if age or years of professional/academic practice influenced such behaviour. This multicentre cross-sectional study was conducted in 2021. In total, 120 nurses from the University Hospital of Pontevedra and 303 nursing students from the Universities of Vigo and Santiago de Compostela (N-W Spain) participated in this study (n = 423). An ad hoc questionnaire was used to evaluate self-medication decision-making. Self-medication for dermatological diseases was reported by 58.39% (n = 247) of participants. Among our respondents, 44.44% of nurses and 42.68% of students would recommend treatment for skin diseases to a third party. We found a higher prevalence of medication without prescription in nurses than in students (*p* < 0.001). More experience (*p* = 0.01) and older age (*p* < 0.001) were associated with more self-medication in the case of nurses and students, respectively. The prevalence of self-medication and treatment recommendation to a third party are cause for concern. Identifying these situations and associated factors may help to implement evidence-based strategies and education.

## 1. Introduction

The World Health Organization defines self-medication (or self-treatment) as the use of medicinal products by consumers to treat diseases or symptoms recognised by the consumers themselves, as well as the intermittent or chronic use of a medication prescribed by a physician for a chronic disease or its symptoms [1]. Self-care is the willingness and ability of people to participate autonomously in and make informed decisions related to their health. Self-medication is included within self-care activities. Self-care is a patient’s own decision, and in many cases, it involves the use of medications without prescription from the healthcare professional responsible for treatment [2].

In these circumstances, it is important to be rational when selecting drugs (appropriate for the disease or symptoms, and with the right doses and duration). Rational drug use may not only give the user a feeling of self-empowerment but also reduce healthcare costs [3]. Regarding both non-prescription and prescription treatments, more than 50% of drugs are used incorrectly. This can lead to diagnostic errors, delays in adequate treatment, worsening and masking of a pathology, or the appearance of adverse pharmacological events [3,4]. Thus, to self-medicate effectively, individuals must be able to recognise their symptoms, medicate themselves appropriately, and know the risks involved [5].

The prevalence of self-medication varies from country to country. It ranges between 46% and 53.3% among adults in general medicine [6]. The prevalence of self-medication in healthcare professionals and their students, including nursing, is troublingly high, despite them being a group that is aware of the potential risks of this practice. This behaviour not only has consequences for the professionals themselves, but also for the patients they care for [7,8,9]. Therefore, it necessary to raise awareness among these professionals about the correct use of medications. They must understand that when they are patients, they must stay within their scope of practice. These measures would help to reduce self-medication and its complications, and subsequently improve rational prescription in daily activities [8,10]. In this regard, a recent review article stressed the need to analyse real behaviours in taking medication and concluded that patient-centred strategies should be considered [11].

A systematic review on dermatology found that self-medication was reported in between 6 and 68% of cases [4]. Furthermore, the accessibility and visibility of mucocutaneous lesions are predisposing factors for the diagnosis and treatment of dermatological pathologies. These factors, together with the apparent but illusive simplicity of their management, can favour self-treatment. However, if the diagnosis or treatment is inadequate, the result of self-treatment may not be as desired and may even be harmful [12].

To the best of our knowledge, there are few studies on self-medication in dermatology, and even fewer cases assess this situation in healthcare professionals, particularly in nurses or future nurses [4,10,13,14]. In this vein, it is necessary to study this specific phenomenon in this professional group.

To this end, the aims of this study were (1) to determine the prevalence of self-medication for skin lesions in nurses and nursing students and (2) to explore how age and professional/academic experience play a part in it.

## 2. Methods

### 2.1. Study Design

A multicentre cross-sectional descriptive study was designed. To ensure adequate data reporting, the STrengthening the Reporting of OBservational Studies in Epidemiology (STROBE) statement was used. This guideline is one of the most widely used in our discipline to report clear and standardised data of observational studies [15].

### 2.2. Participants and Data Collection

The sample consisted of nurses from the University Hospital of Pontevedra and undergraduate nursing students from the Schools of Nursing at the University of Vigo and the University of Santiago de Compostela in northwest Spain. Given their greater knowledge in this field, nurses working in dermatology services were excluded. Participants were recruited through convenience sampling. For this purpose, informative posters were distributed by the researchers, and various methods of communication with possible participants were used.

To calculate the necessary and significant sample size, we began with a target population of 888 nurses and 800 nursing students. Based on these data, with a level of confidence (1 − α) of 95%, a margin of error (d) of 5%, and a proportion or estimated percentage level (*p*) of 90%, 120 valid questionnaires were deemed necessary in the case of nurses and 118 in nursing students. The needed number of participants was finally reached, with a response rate of 13.51% (n = 120/888) in nurses and 37.88% (n = 303/800) in nursing students.

### 2.3. Instrument

Data were collected during the first semester of 2021 using a self-administered, anonymous, and voluntary questionnaire which was created ad hoc for this sociodemographic context. To achieve the aforementioned objectives, a bibliographic review of the medical literature was carried out [4,10,13,14], considering the specific nursing perspective to create the instrument. Due to the nature of the questionnaire, its content and criteria were later validated by experts/judges (10 dermatologists and 10 dermatology nurses). To quantify the responses, Aiken’s V was used, thus improving the final selection of items by seeking a level of agreement greater than a coefficient of 0.70 in a range from 0 to 1 [16,17].

The Forms^®^ application integrated into the Office 365^®^ package was used to complete the questionnaires. This guaranteed the anonymity and confidentiality of the data collected.

The key questions were whether the participants self-treated for any general or specifically dermatological diseases. In those who admitted to self-medication, the following characteristics were collected: route of administration, treatment group, duration of the skin lesions before self-medicating, area of lesions, dermatological condition leading to self-medication, duration of treatment, reason to self-treat, source of information if self-treatment was chosen on one’s own, as well as patient information leaflet and expiration date review. We also recorded whether the dermatological disease resolved after self-medication or whether a dermatologist’s evaluation was necessary (which included medical indications given at this consultation related to treatment and diagnosis). Finally, nurses and nursing students were asked about their self-confidence in recommending a treatment to someone else who presented with a dermatosis like theirs.

In addition to the questions related to self-medicated drug use, the following sociodemographic data were collected: gender, age, years of professional practice, academic year, and practice setting, if applicable.

### 2.4. Data Analysis

Statistical analyses were performed using R Statistics^®^ (Ri 386 3.4.2 version, R commander package). The frequency distribution of the qualitative variables and the mean and standard deviation of the quantitative variables were calculated. To determine the association between qualitative and quantitative variables, the chi-square and Student t-tests were used, respectively. *p* value < 0.05 was considered as the nominal threshold for statistical significance. Odds ratio and Cohen’s d were used to measure the effect size.

### 2.5. Ethical Considerations

The study protocol was approved by the regional Research Ethics Committee of Pontevedra-Vigo-Ourense (N-W Spain) (Approval number 2020/534). At the beginning of the questionnaire, implied consent was obtained according to European and national regulations, where participants confirmed that they comprehensively understood the information and purposes of this study.

## 3. Results

The sample consisted of 423 participants, of whom 120 were nurses and 303 were nursing students. Regarding nurses (n = 120), 90% were females (n = 102), with an average age of 38.64 (SD = 12.50 years). Of the total, 45% (n = 54) had been practicing for 15 years or more (M = 13.62; SD = 12.04 years) in medical services (69.17%; n = 83), followed by medical–surgical (26.67%; n = 32). With respect to nursing students (n = 303), 87.13% (n = 264) were females. The average age was 20.59 (SD = 4.50 years). The majority (56.44%; n = 171) were in their first academic year. Table 1 presents the characteristics of the study participants.

**Figure 1:**
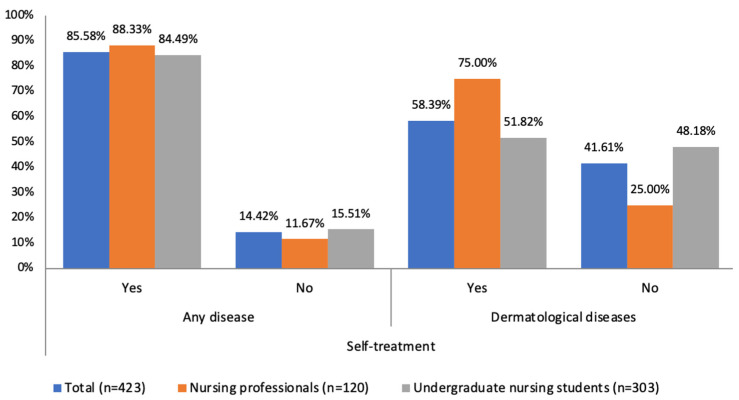
Prevalence of self-treatment without prescription.

In general, 85.58% (n = 362) reported having self-medicated on various occasions in the past. The percentage of respondents who used self-medication for dermatological diseases was 58.39% (n = 247). This prevalence was higher in practicing nurses than in nursing students (88.33% vs. 84.49% regarding self-medication in general and 75.00% vs. 51.82% in the case of dermatological self-medication) (see Figure 1).

### 3.1. Self-Medication in Nurses

The most frequent route of administration for dermatologic conditions was topical (98.89%; n = 89/90), followed by oral (37.78%; n = 34/90). Among topical drugs, corticosteroids (65.52%; n = 57/87) ranked at the top, followed by antifungals (35.63%; n = 31/87), while antihistamines (48.48%; n = 16/33) were the most used oral medication (Table 2). In most cases, the duration of skin lesions prior to self-medicating was less than a month (62.22%; n = 56/90). Their location distribution in covered (53.93%; 48/89) or visible/exposed (47.19%; n = 42/89) areas was similar.

Table 2 shows the frequency distribution of the different dermatoses where self-medication was used. Contact dermatitis (35.56%; n = 32/90) and fungal infections (28.89%, n = 26/90) stood out. In 66.67% of the cases (n = 60/90), the treatment was usually maintained until the lesions resolved.

**Figure 2:**
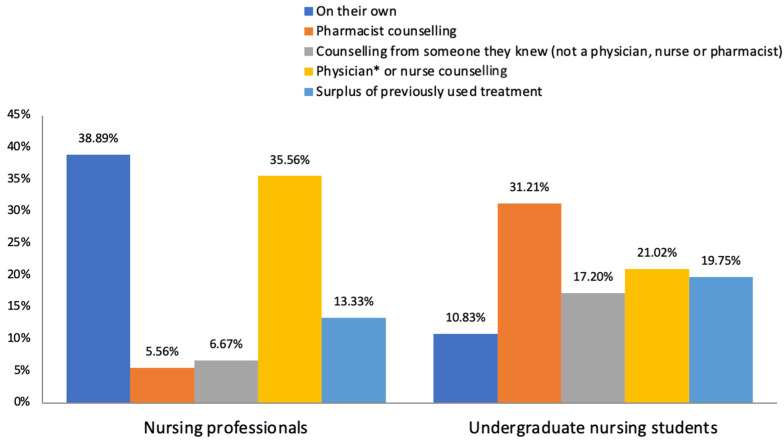
Factors influencing dermatological self-medication. * Not a dermatologist.

Most nurses (38.89%; n = 35/90) decided to self-treat on their own initiative (see Table 3). Of these, the majority said that they relied on the knowledge acquired during their undergraduate training period (81.25%; n = 26/32). Others self-treated on the advice of a medical professional (not a dermatologist) or another nurse (35.56%; n = 32/90) (Figure 2).

Regarding medication, 93.33% (n = 84/90) of professionals read the information regarding the expiration date on the products, whereas 81.11% (n = 73/90), 74.44% (n = 67/90), and 77.78% (n = 70/90) reviewed details on their dosage, side effects, and contraindications, respectively, in the patient information leaflet.

A resolution of the pathology was reported in 86.67% (n = 78/90) after self-medication, whereas 15.56% (n = 14/90) needed a dermatology consultation. In 28.57% (n = 4/14), the self-medication was not adequate and in 50% (n = 7/14), another treatment was indicated. The correlation between the diagnosis issued by a dermatologist and the nurses’ suspicions was 78.57% (n = 11/14).

Forty-four percent (n = 40/90) of the professionals who used treatment without a medical prescription said they would advise someone else on what treatment to apply if they presented with a dermatosis like theirs.

### 3.2. Self-Medication in Nursing Students

The most frequently used route of administration of dermatological diseases was topical (100%, n = 157). Among topical drugs, corticosteroids (35.53%, n = 54/152) ranked at the top, followed by antibiotics (21.05%, n = 32/152). The oral route accounted for 37.58% (n = 59/157) of cases. The most frequent oral drugs were antihistamines (19.30%, n = 11/57) (see Table 2). The skin lesions that led to self-medication were characterised by an evolution time of less than 1 month (37.58%; n = 59/157) or more than one year (27.39%, n = 43/157), and were usually located in visible areas (70.06%; n = 110/157).

Acne was the most commonly self-medicated condition (40.13%; n = 63/157). Contact dermatitis ranked second (21.02%; n = 33/157) (Table 2). The most common procedure was to continue with the treatment until the lesion resolved (59.24%; n = 93/157).

Advice from a pharmacist was the main reason which led to the decision to self-medicate (31.21%; n = 49/157), followed by advice from a physician (not a dermatologist) or another nurse (21.02%; n = 33/157). Others stated that the reason was having a surplus of previous medications at home (n = 19.75%; n = 31/157) (Figure 2). A supposed knowledge about the disease and its treatment was the most frequent reason to self-treat on their own initiative (58.82%; n = 10/17). The sources of information that influenced participants’ own decision-making are shown in Table 3.

More than half of the time, students consulted the product to find out about its expiration date (77.71%; n = 122/157), as well as learned about the dosage (59.87%, n = 94/157), side effects (55.41%; n = 87/157), and contraindications (54.78%; n = 86/157) in the patient information leaflet.

Eighty-six percent (n = 136/157) of students considered that the self-administered treatment resolved their dermatosis, while 14.65% (n = 23/157) consulted a dermatologist. Of these, 39.13% (n = 9/23) of them were told that the self-medication was not adequate, and the majority had their treatment changed (69.57%; n = 16/23). The concordance between the diagnosis suspected by the students and that given by the dermatologists was 86.96% (n = 20/23).

Forty-two percent (67/157) of the students reported that they would advise another person on what treatment to apply if they suffered from a similar condition.

### 3.3. Influence of Age and Professional/Academic Career on Self-Medication in Nursing

When looking at the relationship between medication use without a legitimate prescription and sociodemographic profiles, there was a statistically significant connection between the dermatologic conditions and the duration of years of professional practice (*p* = 0.01) (see Table 4 for more details).

Older nursing students self-medicated more frequently for both all (*p* = 0.014) and dermatologic conditions (*p* < 0.001). However, there was no association with academic year (Table 5).

Furthermore, the prevalence of self-medication for dermatological diseases was significantly higher in nurses than in students (OR 2.09, *p* < 0.001).

## 4. Discussion

Numerous studies have evaluated the prevalence of self-medication in the general population or specific groups of nursing and nursing students. Nevertheless, to the best of our knowledge, there are no studies analysing this incidence in dermatology. In this vein, this research investigates the nature of this phenomenon and the association of self-medication with age and academic/professional career.

It has been documented that self-medication among nurses, as well as other healthcare professionals, is more frequent than in the general population [3,7,18]. Although published research has mainly focused on nurses, there are numerous reports that measure the occurrence of self-medication in various healthcare professionals jointly, such as physicians, nursing assistants, technicians, or pharmacists. They report that between 24.2% and 73.4% resort to self-medication [3,8]. Analgesics, along with anti-inflammatories and antibiotics, remain the top three pharmacological groups [3,8,19]. When the percentage of self-medication of certain drugs, such as antibiotics, is evaluated, it is between 60.4% and 70.8% [7,20].

Data on the relationship between age, professional experience, and self-medication are conflicting [3]. Some report higher incidences of self-medication among younger and less experienced individuals. Unlike the data found in the literature regarding general self-medication [8,19], our study revealed that practical experience plays a key role in professional judgement when considering dermatological self-medication.

Similarly, it has been documented that the prevalence of self-medication in health sciences students ranges from 38.0% to 97.8%, depending on the country of origin, the students, the degree, or the period of time used for evaluation [9,21,22,23,24,25,26,27,28,29].

A study of nursing students conducted in Spain between 2016 and 2017 is worth mentioning. The study stated that 73.8% had self-medicated in the previous month, and analgesics had predominantly been used (88.91%) [2]. Other research observed a similar or an even higher prevalence of self-medication (76–92%) [24,27,28,29]. Moreover, the percentage of self-medication among nursing and medical students was analysed in specific pathologies such as dysmenorrhea (65%) [30] or musculoskeletal pain (59.9%) [31], as well as with specific drugs, such as antibiotics (52.7%) [9] or analgesics (very different prevalences between studies, ranging from 39% [32,33] to 87% [21]).

In line with our results, other researchers have found a significant association of older age and longer duration of practice/studies with a higher frequency of self-medication [33].

In our study, we found a high prevalence of self-medication for any pathology, both in nurses and students (88.33% and 84.49%, respectively), as well as in self-medication for dermatological diseases, although this shows lower figures (75% and 51.82%, respectively). Due to the scarcity of studies assessing these last data, we cannot establish comparisons.

The differences found in the decisions to self-medicate, in addition to characteristics inherent to each population, can be explained by the fact that there are several studies that limited the time in which self-medication was administered to the days or months immediately prior to the questionnaire [2,8,19]. Thus, it is expected that the shorter the period of time evaluated, the lower the prevalence will be.

Drugs were obtained at work or in clinical practice settings in a non-negligible percentage of cases. These data are alarming, as they reflect a lack of control in the dispensing of medication within healthcare services, which should be the ideal place to inculcate the rational use of medications [29].

Concerning the areas affected by the participating nursing students, a higher frequency of self-medication was detected if skin lesions were visible. Given the lack of studies focused on self-medication for skin lesions, it is not possible to compare these data. However, it seems reasonable to assume that greater lesion exposure creates the need for rapid relief and leads to people looking for immediate treatment.

Among the reasons leading to self-medication, the current literature lists the following: reuse of previous prescriptions, sharing medications with family and friends due to a surplus of drugs at home, or advice from pharmacists and pharmacy assistants. Advertisements, the excess of information on the internet, and ease of access in pharmacies also favour self-medication [4]. In the case of nurses and/or nursing students, the knowledge and experience acquired over years of practice or while studying in the field equips them with knowledge on medications, including their pharmacological actions, indications, contraindications, and possible adverse effects [2,3,9]. Likewise, it has been described that being overworked, caring for seriously ill patients, emotional labilities such as stress, and ease of access to medications are additional factors contributing to self-medication [3,19]. To this list, other research adds the lack of time, privacy issues, consulting with various specialists, delayed access, or disease severity [2,7,8,29].

Self-medication has also been associated with a lack of awareness about its negative implications [29]. In this sense, a study among healthcare professionals, who were predominantly nurses, showed that 46.7% considered that self-medication with antibiotics was a safe practice and 52.4% believed that they could successfully treat common infectious diseases independently [7]. Along the same lines, Ali and cols. (2021) [20] found that despite claiming to rely on their own knowledge on the use of antibiotics, only 20.7% of nurses completed the antibiotic regimen correctly.

Our results showed that more than 74% of nurses and more than 54% of students consulted patient information leaflets. Similar numbers have been found both in nurses and assistants (76.9%) [3] as well as nursing students (64.24%) [2] who consulted leaflets before self-medicating.

In relation to recommending medication to third parties, a study conducted among nursing students in another Spanish region revealed that 66.4% would recommend a treatment to an individual with similar symptoms [2]. In our study, 44.44% of nurses and 42.68% of students would make this recommendation. This fact seems to be related to knowledge about diseases and medications, which creates a false confidence not only in self-medication, but also in recommending drugs to others. However, this promotion of self-medication to a third party exceeds the professional role and can put the health of other people at risk [34]. Furthermore, healthcare professionals can expect that others may seek advice on medications or medical conditions and even ask for recommendations even when a specialist is clearly necessary [34].

According to our data, the number of participants who required a subsequent dermatological consultation (15.56% of nurses and 14.65% of students) is similar to the 17.1% observed in another study carried out on nurses and assistants [3]. Although the diagnostic correlation between the participants and the dermatologists was high (>75%), no conclusions can be drawn since the number of participants who finally went to the dermatologist was low. This issue should also be studied in future research.

### 4.1. Strengths and Limitations

To the best of our knowledge, this study is the first to explore self-medication in these professional groups. Therefore, it contributes to filling the gap in research on dermatological self-medication in healthcare professionals and to establishing measures for students and during subsequent professional development. Some limitations of this study should be noted, nonetheless. As the questionnaire was completed retrospectively, it may have contributed to memory bias, which could lead to a falsely low prevalence in the results. However, documenting the practice of self-medication at any previous moment, without time constraints, favours a more realistic prevalence in comparison with other studies that set time limits.

### 4.2. Conclusion and Practice Implications

General and dermatological self-medication in nurses and nursing students is high. Recommending treatment to a third party is a cause for concern. Nurses and nursing students play an important role in administering medication as well as in patient safety. Having identified the rationales for and influences on dermatological self-medication may help promote measures to stop such practices. Ongoing education, training, and coaching are necessary to guarantee safe and efficient care for all those involved in studying, providing, and receiving care. In this sense, it is vital to improve the health literacy skills of nurses and future nurses so that they can identify the dangers and their own competences in this regard.

## Figures and Tables

**Table 1:** Sociodemographic characteristics of nurses and nursing students who self-medicated.

**Nurses**	**Self-medication**		**For any disease (n = 106)**		**For dermatological diseases (n = 90)**	
			*Mean*	*SD*	*Mean*	*SD*
	**Age (years)**		38.91	±12.65	39.62	±12.36
	**Years of practice**		14.03	±12.09	15.41	±12.20
			*Frequency* *(n)*	*Percentage* *(%)*	*Frequency* *(n)*	*Percentage* *(%)*
	**Gender**	Male	11	10.38	7	7.78
		Female	95	89.62	83	92.22
	**Practice** **setting**	MD	73	68.87	60	66.67
		S	4	3.77	4	4.44
		MD-S	29	27.36	26	28.89
**Nursing students**	**Self-medication**		**For any disease (n = 256)**		**For dermatological diseases (n = 157)**	
			*Mean*	*SD*	*Mean*	*SD*
	**Age (years)**		20.75	±4.82	21.45	±5.91
			*Frequency* *(n)*	*Percentage* *(%)*	*Frequency* *(n)*	*Percentage* *(%)*
	**Gender**	Male	32	12.50	17	10.83
		Female	224	87.50	140	89.17
	**Academic year**	First	149	58.20	86	54.78
		Second	62	24.22	42	26.75
		Third	36	14.06	22	14.01
		Fourth	9	3.52	7	4.46

MD: medical; S: surgical; SD: standard deviation.

**Table 2:** Route of administration, treatment group, and skin diseases treated with self-medication.

		**Nurses (n = 87)**		**Nursing students (n = 152)**	
		*Frequency* *(n)*	*Percentage* *(%)*	*Frequency* *(n)*	*Percentage* *(%)*
**Topical treatment**	**Antifungal**	31	35.63	18	11.84
	**Antibiotic**	26	29.89	32	21.05
	**Corticosteroid**	57	65.52	54	35.53
	**Retinoid**	5	5.75	2	1.32
	**Antihistamine**	20	22.99	17	11.18
	**Corticosteroid + antifungal**	12	13.79	1	0.66
	**Corticosteroid + antibiotic**	10	11.49	7	4.61
	**Other**	3	3.45	1	0.66
	**Could not remember**	3	3.45	3	1.97
		**Nurses (n = 33)**		**Nursing students (n = 57)**	
		*Frequency* *(n)*	*Percentage* *(%)*	*Frequency* *(n)*	*Percentage* *(%)*
**Oral treatment**	**Antifungal**	10	10.30	4	7.02
	**Antibiotic**	6	18.18	7	12.28
	**Corticosteroid**	6	18.18	2	3.51
	**Antihistamine**	16	48.48	11	19.30
	**Other**	3	9.09	3	5.26
	**Not remembered**	1	3.03	36	63.16
		**Nurses (n = 90)**		**Nursing students (n = 157)**	
		*Frequency* *(n)*	*Percentage* *(%)*	*Frequency* *(n)*	*Percentage* *(%)*
**Skin diseases**	**Acne**	11	12.22	63	40.13
	**Psoriasis**	3	3.33	7	4.46
	**Atopic dermatitis**	17	18.89	1	0.64
	**Allergic or irritative contact dermatitis**	32	35.56	33	21.02
	**Seborrheic dermatitis**	8	8.89	8	5.1
	**Other eczemas**	8	8.89	27	17.20
	**Melanocytic nevi**	0	0.00	1	0.64
	**Urticaria**	5	5.56	13	8.28
	**Fungal infections**	26	28.89	9	5.73
	**Bacterial infections**	10	11.11	7	4.46
	**Parasitic infections**	4	4.44	2	1.27
	**Warts**	3	3.33	2	1.27
	**Sexually transmitted diseases**	0	0.00	0	0.00
	**Insect bites**	21	23.33	27	17.20
	**Skin burns**	20	22.22	25	15.92
	**Other**	4	4.44	9	5.73
	**Not remembered**	0	0	1	0.64
	**Unknown diagnosis**	2	2.22	11	7.01

**Table 3:** Source of information if self-treatment was chosen on one’s own.

	Nurses (n = 35)		Nursing Students (n = 17)	
	*Frequency* *(n)*	*Percentage* *(%)*	*Frequency* *(n)*	*Percentage* *(%)*
**Own knowledge about the disease and its treatment**	32	91.43	10	58.82
**Medicine books/journals**	6	17.14	1	5.88
**Internet**	3	8.57	6	35.29
**Advertisement on television**	0	0.00	1	5.88
**Other**	2	5.71	0	0.00

**Table 4:** Association between professional practice and self-treatment in nurses.

Self-Medication			For any Disease (n = 120)			For Dermatological Diseases (n = 106)		
			*Mean*	*p value*		*Mean*	*p value*	
**Average years of professional practice**		Yes	14.02	0.304 *		15.41	<0.001 *	
		No	10.50			6.25		
						Cohen’s d = 0.895		
			*Frequency* *(n)*	*Percentage* *(%)*	*p value*	*Frequency* *(n)*	*Percentage* *(%)*	*p value*
**Number of nurses (%) by years of professional practice**		<5 years	39	36.80	*p* = 0.1681 ^#^	28	31.1	*p* = 0.014 ^#^
		5–15 years	17	16.00		15	16.70	
		>15 years	50	47.20		47	52.20	
						>15 years vs. <5 years: OR 6.15 (1.58–23.97) >15 years vs. 5–15 years: OR 2.09 (0.32–13.71)		

Statistical tests for comparisons: * Student’s *t*-test, ^#^ chi-square test.

**Table 5:** Association between age and self-treatment in nursing students.

Self-Medication		For Any Disease (n = 303)		For Dermatological Diseases (n = 256)	
		*Mean*	*p value*	*Mean*	*p value*
**Average age (years)**	Yes	20.75	0.014 *	21.45	<0.001 *
	No	19.74		19.63	
		Cohen’s d = 0.277		Cohen’s d = 0.419	

Statistical test for comparisons: * Student’s *t*-test.

## Data Availability

The data that support the findings of this study are available from the corresponding author upon reasonable request.

## References

[1] WHO_EDM_QSM_00.1_eng.pdf. https://apps.who.int/iris/bitstream/handle/10665/66154/WHO_EDM_QSM_00.1_eng.pdf?sequence=1&isAllowed=y.

[2] Andrés M.I.G., Blanco V.G., Verdejo I.C., Guerra J.A.I., García D.F. (2021). Self-Medication of Drugs in Nursing Students from Castile and Leon (Spain). Int. J. Environ. Res. Public. Health..

[3] De Borrajo Lama C., Arribas A.A. (2004). Self medication in nursing. Rev. Enferm. Barc. Spain..

[4] Corrêa-Fissmer M., Mendonça M.G., Martins A.H., Galato D. (2014). Prevalence of self-medication for skin diseases: A systematic review. An. Bras. Dermatol..

[5] Calamusa A., Di Marzio A., Cristofani R., Arrighetti P., Santaniello V., Alfani S., Carducci A. (2012). Factors that influence Italian consumers’ understanding of over-the-counter medicines and risk perception. Patient Educ. Couns..

[6] de Loyola Filho A.I., Uchoa E., Guerra H.L., Firmo J.O.A., Lima-Costa M.F. (2002). Prevalência e fatores associados à automedicação: Resultados do projeto Bambuí. Rev. Saúde Pública.

[7] Kassa T., Gedif T., Andualem T., Aferu T. (2022). Antibiotics self-medication practices among health care professionals in selected public hospitals of Addis Ababa. Ethiopia. Heliyon.

[8] Fekadu G., Dugassa D., Negera G.Z., Woyessa T.B., Turi E., Tolossa T., Fetensa G., Assefa L., Getachew M., Shibiru T. (2020). Self-Medication Practices and Associated Factors Among Health-Care Professionals in Selected Hospitals of Western Ethiopia, *Patient Prefer*. Adherence.

[9] Janatolmakan M., Abdi A., Andayeshgar B., Soroush A., Khatony A. (2022). The Reasons for Self-Medication from the Perspective of Iranian Nursing Students: A Qualitative Study. Nurs. Res. Pract..

[10] Karamata V.V., Gandhi A.M., Patel P.P., Desai M.K. (2017). Self-medication for Acne among Undergraduate Medical Students. Indian J. Dermatol..

[11] Mullen R.J., Duhig J., Russell A., Scarazzini L., Lievano F., Wolf M.S. (2018). Best-practices for the design and development of prescription medication information: A systematic review. Patient Educ. Couns..

[12] Battyáni Z. (2006). Dermatological treatment. Orv. Hetil..

[13] Tameez-Ud-Din A., Malik I.J., Bhatti A.A., Din A.T.U., Sadiq A., Khan M.T., Chaudhary N.A., Arshad D., Knowledge A.O. (2019). Attitude, and Practices Regarding Self-medication for Acne Among Medical Students. Cureus.

[14] Kombaté K., Técléssou J.N., Saka B., Akakpo A.S., Tchangai K.O., Mouhari-Toure A., Mahamadou G., Gnassingbé W., Abilogun-Chokki A., Pitché P. (2017). Prevalence and Factors Associated with Self-Medication in Dermatology in Togo. Dermatol. Res. Pract..

[15] Von Elm E., Altman D.G., Egger M., Pocock S.J., Gøtzsche P.C., Vandenbroucke J.P. (2008). The Strengthening the Reporting of Observational Studies in Epidemiology (STROBE) statement: Guidelines for reporting observational studies. J. Clin. Epidemiol..

[16] Sireci S., Faulkner-Bond M. (2014). Validity evidence based on test content. Psicothema.

[17] Sampieri R.H., Fernandez-Collado C.F. (2014). Metodología de la Investigación, Sexta Edición.

[18] Sharif S.I., Bugaighis L.M.T., Sharif R.S. (2015). Self-Medication Practice among Pharmacists in UAE. Pharmacol. Amp Pharm..

[19] Barros A.R.R., Griep R.H., Rotenberg L. (2009). Self-medication among nursing workers from public hospitals. Rev. Lat. Am. Enfermagem..

[20] Ali A.S., Jandani R., Al-Qahtani A.A., Alenzi A.A.S. (2021). Preliminary findings of a study on the practice of self-medication of antibiotics among the practicing nurses of a tertiary care hospital. J. Taibah Univ. Med. Sci..

[21] Al-Hussaini M., Mustafa S., Ali S. (2014). Self-medication among undergraduate medical students in Kuwait with reference to the role of the pharmacist. J. Res. Pharm. Pract..

[22] Kumar N., Kanchan T., Unnikrishnan B., Rekha T., Mithra P., Kulkarni V., Papanna M.K., Holla R., Uppal S. (2013). Perceptions and practices of self-medication among medical students in coastal South India. PLoS ONE.

[23] Lukovic J.A., Miletic V., Pekmezovic T., Trajkovic G., Ratkovic N., Aleksic D., Grgurevic A. (2014). Self-Medication Practices and Risk Factors for Self-Medication among Medical Students in Belgrade, Serbia. PLoS ONE.

[24] Goel D. (2013). Self-medication patterns among nursing students in North India. IOSR J. Dent. Med. Sci..

[25] de Aquino D.S., de Barros J.A.C., da Silva M.D.P. (2010). A automedicação e os acadêmicos da área de saúde. Ciênc. Saúde Coletiva..

[26] Incidência da Automedicação em Graduandos de Enfermagem, Repositório Digit. UNIP. https://repositorio.unip.br/journal-of-the-health-sciences-institute-revista-do-instituto-de-ciencias-da-saude/incidencia-da-automedicacao-em-graduandos-de-enfermagem/.

[27] Azodo C., Ehigiator O., Ehigiator L., Ehizele A., Ezeja E., Madukwe I. (2013). Self-medication practices among dental, midwifery and nursing students. Eur. J. Gen. Dent..

[28] Johnson D., Sekhar H.S., Alex T., Kumaraswamy M., Chopra R.S. (2016). Self medication practice among medical, pharmacy and nursing students. Int. J. Pharm. Pharm. Sci..

[29] Gama A.S.M., Secoli S.R. (2017). Self-medication among nursing students in the state of Amazonas—Brazil. Rev. Gaucha Enferm..

[30] Bharati J.P., Ulak S., Shrestha M.V., Dixit S.M., Acharya A., Bhattarai A. (2021). Self-medication in Primary Dysmenorrhea among Medical and Nursing Undergraduate Students of a Tertiary Care Hospital: A Descriptive Cross-sectional Study. JNMA J. Nepal Med. Assoc..

[31] Martinez J.E., Pereira G.A.F., Ribeiro L.G.M., Nunes R., Ilias D., Navarro L.G.M. (2014). Study of self-medication for musculoskeletal pain among nursing and medicine students at Pontifícia Universidade Católica—São Paulo. Rev. Bras. Reumatol..

[32] Souza L.A.F., da Silva C.D., Ferraz G.C., Sousa F.A.E.F., Pereira L.V. (2011). The prevalence and characterization of self-medication for obtaining pain relief among undergraduate nursing students. Rev. Lat. Am. Enfermagem..

[33] Faqihi A.H.M.A., Sayed S.F. (2021). Self-medication practice with analgesics (NSAIDs and acetaminophen), and antibiotics among nursing undergraduates in University College Farasan Campus, Jazan University, KSA. Ann. Pharm. Fr..

[34] Soroush A., Abdi A., Andayeshgar B., Vahdat A., Khatony A. (2018). Exploring the perceived factors that affect self-medication among nursing students: A qualitative study. BMC Nurs..

